# Can the COVID-19 Vaccine Cause Recrudescence of Herpes Zoster Virus While Taking Antiviral Medication?

**DOI:** 10.7759/cureus.38040

**Published:** 2023-04-24

**Authors:** Ameije Ismaili, Stefan Anthony, Jessica Clark

**Affiliations:** 1 College of Osteopathic Medicine, Lake Erie College of Osteopathic Medicine, Bradenton, USA; 2 Dermatology, Coastal Skin Surgery and Dermatology, Panama City, USA

**Keywords:** herpetic neuralgia, shingles complications, varicella-zoster virus, herpes zoster reactivation, covid 19

## Abstract

Coronavirus disease 2019 (COVID-19) represents a multisystem disease that has caused a devastating global pandemic. The COVID-19 vaccine produced in response to the pandemic has been effective but can have side effects. One well-established condition is the reactivation of herpes zoster (HZ). Various risk factors increase the risk of HZ reactivation such as age, infections, and immunosuppressed states. HZ can have severe complications, including herpes zoster ophthalmicus and postherpetic neuralgia. Here, we present a unique case where a patient experienced HZ reactivation after both primary doses of the COVID-19 vaccine despite receiving early antiviral treatment.

## Introduction

Primary infection by the neurotropic varicella-zoster virus (VZV) or human herpes virus 3 (HHV-3) causes chickenpox while secondary reactivation of VZV causes shingles or herpes zoster (HZ). VZV infects the mucous membranes, skin, and neurons. It can invade the immune system and establish a latent infection in the sensory ganglia. Common risk factors for HZ reactivation are age >50 years, infections, stress, and immunosuppression [1]. Latent infection can commonly occur in the trigeminal sensory ganglia. Reactivation causes acute neuralgia and the evolution of a characteristically erythematous, then maculopapular rash, followed by vesicular rash involving the trigeminal nerve distribution [2]. One characteristic presentation is herpes zoster ophthalmicus causing dermatologic pathologies and pain in the trigeminal nerve V1 ophthalmic nerve distribution. V1 is critical, as it innervates the cornea, and in severe chronic herpes zoster ophthalmicus, a patient is at risk of blindness [2].

Reactivation of HZ is now a known potential adverse event of the COVID-19 vaccine [1]. Given the risk factor profile of HZ reactivation, it is clear how the causal link between the COVID-19 vaccine and the risk of HZ can be made. It has been reported that HZ reactivation can occur following inactivated influenza, hepatitis A, rabies, and Japanese encephalitis vaccines [3]. Here, we report a case of shingles involving trigeminal nerve distribution appearing after the first dose of the COVID-19 vaccine and recurring after the second dose of the COVID-19 vaccine, even while receiving valacyclovir therapy.

## Case presentation

**Figure 1:**
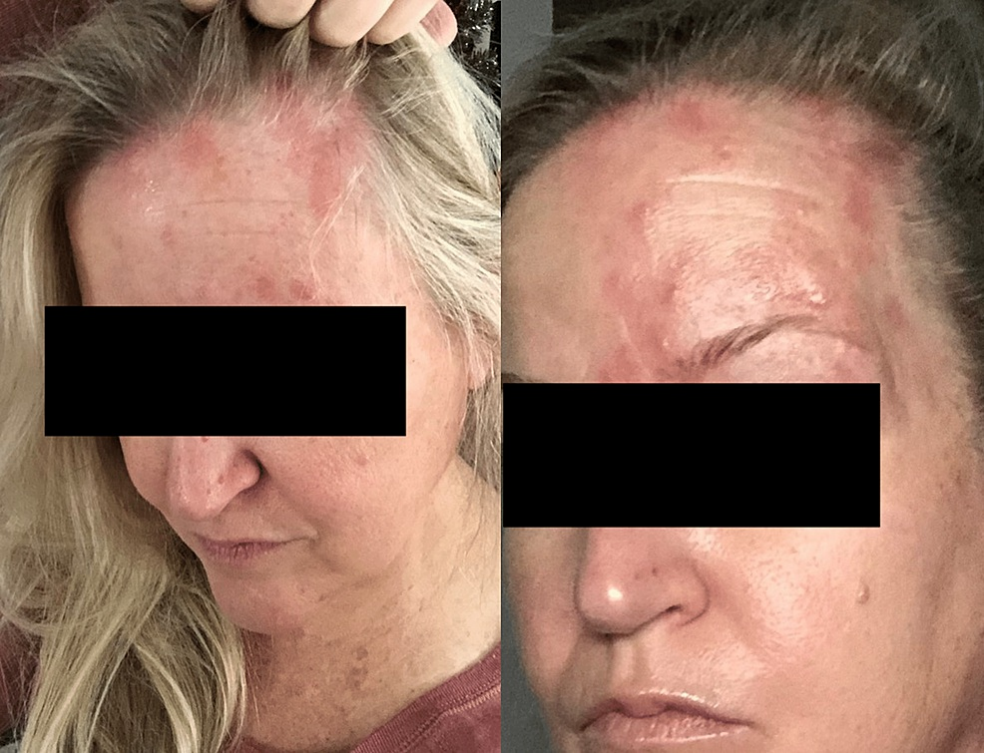
The patient one week after the first dose of the COVID-19 vaccine when she began experiencing shingles

A 51-year-old female presented to an outpatient dermatology clinic due to a rash on her face and left eyelid. The rash is blistering, burning, and severe. The rash has been present for two weeks. She experienced chills, fever, and a sore throat for three days. The patient had recently received a first-dose Moderna COVID-19 vaccine. One week after receiving the vaccine, the patient broke out with shingles on the left side of her face, inner eye, throat, ear, and neck (Figure 1).

**Figure 2:**
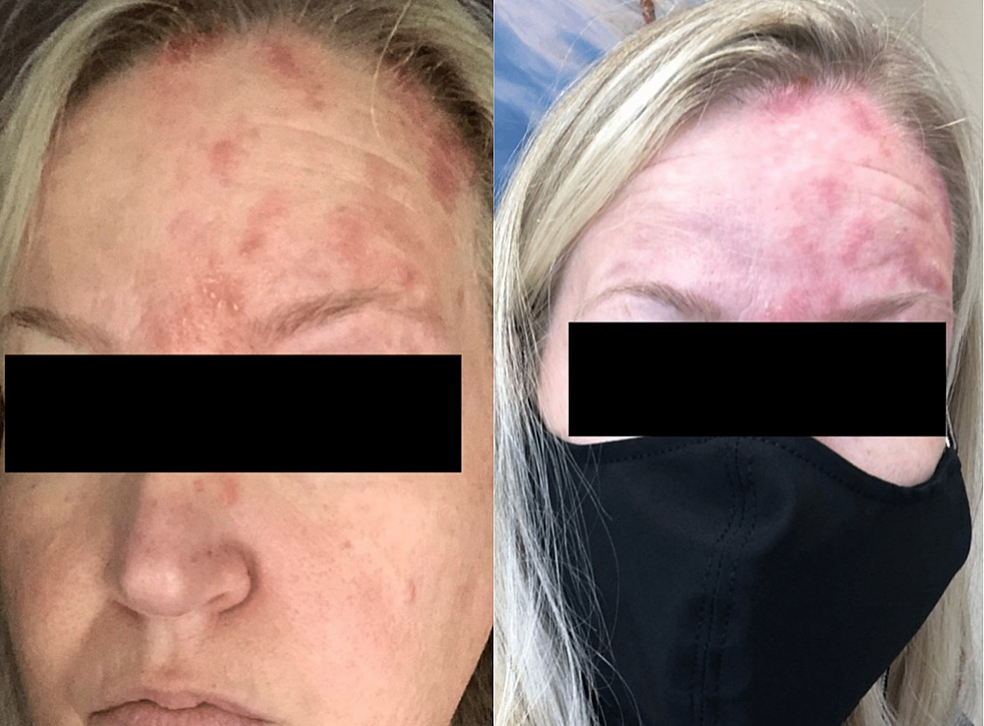
The patient after the second dose of the COVID-19 vaccine

The patient did have chickenpox as a child. She was advised to take valacyclovir until after the second COVID-19 vaccine and gabapentin as needed. After receiving the second dose of the COVID-19 vaccine, she experienced an immediate shingles reaction despite taking valacyclovir (Figure 2).

**Figure 3:**
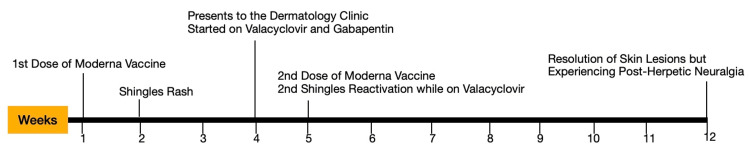
Case presentation timeline in weeks

Shingles affected the left eye, face, scalp, ear, neck, and throat. The patient had little immediate relief from valacyclovir or oral prednisone taper. Over the next three months, the patient experienced resolution of skin lesions with fading hyperpigmentation still present, however, she is still experiencing pain in the V1 distribution consistent with postherpetic neuralgia. The patient does not have diabetes mellitus or any other known immunodeficiency. However, her past medical history includes atrial fibrillation, hypothyroidism, endometriosis, and deep vein thrombosis. For clarity, we have provided a timeline of the vaccinations and events described in this case presentation (Figure 3).

## Discussion

In this case report, we present a middle-aged female who experienced moderate-severe herpes zoster in trigeminal nerve distribution after receiving the COVID-19 vaccine. Reactivation of latent VZV can occur in the sensory ganglia. Risk factors for reactivation include increasing age, infections, stress, and immunosuppression [1]. Our patient’s age and vaccination likely increased her risk of HZ reactivation. Generally, VZV reactivation occurs due to a failure of the T lymphocyte to control the latent infection. Clearly, it is understood how the risk factors of HZ reactivation can predispose individuals to this susceptible state. Furthermore, it has been postulated that exposure to COVID-19 vaccination can act as an immune system stressor producing a transient immunosuppressive state or impaired functioning of T lymphocytes predisposing individuals to HZ reactivation [4-5].

One study analyzing the U.S. Vaccine Adverse Event Reporting System (VAERS) database found 5,934 reported cases of HZ after the COVID-19 vaccine [6]. Of note, they reported that 90% were determined “non-serious” cases with an estimated incidence of 0.7/100,000 cases [6]. The limited severity and low incidence point to HZ reactivation likely being low risk for those receiving the COVID-19 vaccine.

Beyond the dermatologic and painful symptomatology of HZ reactivation, there exist many complications that can arise. One of the most common complications is postherpetic neuralgia which our patient experienced. Of note, she experienced this complication despite receiving prompt antiviral treatment. Postherpetic neuralgia is defined as persistent pain four to nine weeks after the onset of the rash [1]. Our patient also suffered from herpes zoster ophthalmicus, which is defined as HZ involvement of the ophthalmic division of the fifth cranial nerve [7]. Ocular involvement is a serious consequence of herpes zoster ophthalmicus and puts individuals at risk of blindness. Early diagnosis and treatment are critical in decreasing the risk of blindness [7]. Other complications include Ramsay Hunt syndrome, acute retinal necrosis, aseptic meningitis, encephalitis, Guillain-Barré Syndrome, and secondary bacterial infection [8].

In order to limit the severity of HZ primary disease and its complications, early diagnosis and treatment are key. The standard antiviral treatment for HZ is acyclovir. However, resistance to acyclovir can occur, typically via mutations in viral thymidine kinase or DNA polymerase [1]. In cases where resistance to acyclovir is seen, famciclovir is another antiviral that is effective in treating HZ [1]. Vaccination against HZ remains the standard of care to prevent reactivation in older adults. There are currently two HZ vaccines for immunocompetent older adults, the Zostavax vaccine, which is a live attenuated vaccine, and the Shingrix vaccine, which is a recombinant adjuvanted glycoprotein subunit vaccine [1]. Lastly, the management of postherpetic neuralgia involves pharmacologic treatment with gabapentin or pregabalin, tricyclic antidepressants (TCAs), and selective serotonin reuptake inhibitors (SSRIs) [1].

## Conclusions

In summary, we present a case of a 51-year-old female who was not immunocompromised and experienced reactivation of HZ after the first and second doses of the COVID-19 vaccination with complications of postherpetic neuralgia. She experienced all these complications despite receiving prompt antiviral therapy. While the value of vaccines and the value of the COVID-19 vaccine has been well demonstrated, it is important to consider and document vaccine-related complications. Additionally, healthcare providers should use caution when administering additional vaccinations when patients have experienced complications after initial doses.
